# Delayed Onset of Positive Feedback Activation of Rab5 by Rabex-5 and Rabaptin-5 in Endocytosis

**DOI:** 10.1371/journal.pone.0009226

**Published:** 2010-02-16

**Authors:** Huaiping Zhu, Hong Qian, Guangpu Li

**Affiliations:** 1 Department of Biochemistry and Molecular Biology, University of Oklahoma Health Sciences Center, Oklahoma City, Oklahoma, United States of America; 2 Department of Applied Mathematics, University of Washington, Seattle, Washington, United States of America; Mount Sinai School of Medicine, United States of America

## Abstract

**Background:**

Rabex-5 is a guanine nucleotide exchange factor (GEF) that specifically activates Rab5, i.e., converting Rab5-GDP to Rab5-GTP, through two distinct pathways to promote endosome fusion and endocytosis. The direct pathway involves a pool of membrane-associated Rabex-5 that targets to the membrane via an early endosomal targeting (EET) domain. The indirect pathway, on the other hand, involves a cytosolic pool of Rabex-5/Rabaptin-5 complex. The complex is recruited to the membrane via Rabaptin-5 binding to Rab5-GTP, suggesting a positive feedback mechanism. The relationship of these two pathways for Rab5 activation in the cell is unclear.

**Methodology/Principal Findings:**

We dissect the relative contribution of each pathway to Rab5 activation via mathematical modeling and kinetic analysis in the cell. These studies show that the indirect pathway constitutes a positive feedback loop for converting Rab5-GDP to Rab5-GTP on the endosomal membrane and allows sensitive regulation of endosome fusion activity by the levels of Rab5 and Rabex-5 in the cell. The onset of this positive feedback effect, however, contains a threshold, which requires above endogenous levels of Rab5 or Rabex-5 in the cell. We term this novel phenomenon “delayed response”. The presence of the direct pathway reduces the delay by increasing the basal level of Rab5-GTP, thus facilitates the function of the Rabex-5/Rabaptin-5-mediated positive feedback loop.

**Conclusion:**

Our data support the mathematical model. With the model's guidance, the data reveal the affinity of Rabex-5/Rabaptin-5/Rab5-GTP interaction in the cell, which is quantitatively related to the Rabex-5 concentration for the onset of the indirect positive feedback pathway. The presence of the direct pathway and increased Rab5 concentration can reduce the Rabex-5 concentration required for the onset of the positive feedback loop. Thus the direct and indirect pathways cooperate in the regulation of early endosome fusion.

## Introduction

Rabex-5 is a guanine nucleotide exchange factor (GEF) for activation of Rab5 [1], a small GTPase that is associated with early endosomal membrane and regulates early endosome fusion and endocytosis [2]–[5]. Rabex-5 knockout mice die early and develop severe skin inflammation [6], suggesting a non-redundant function *in vivo*. Mast cells isolated from Rabex-5 knockout mice show enhanced IgE receptor-mediated degranulation and cytokine release and these effects are due to the loss of Rabex-5 GEF activity for Rab5 [7]. The core GEF domain of Rabex-5 consists of a tandem helical bundle (HB) domain and Vps9 domain [8]. An early endosomal targeting (EET) domain overlaps with the GEF domain and contains the HB domain and an upstream membrane-binding motif (MBM) [9]. The EET domain is essential for targeting of Rabex-5 to early endosomes and activation of Rab5 in the cell. In addition to this EET-mediated direct membrane targeting, Rabex-5 can also form a complex with Rabaptin-5 and indirectly target to early endosomes via the binding of Rabaptin-5 to Rab5-GTP [10], [11]. Rabex-5 binds to Rabaptin-5 via a coiled-coil region downstream of the Vps9 domain [7], [9], [12], [13].

Both direct and indirect membrane targeting pathways allow Rabex-5 to associate with the membrane and to interact efficiently with its membrane-bound substrate, Rab5-GDP. However, relative contribution of each pathway to the Rabex-5 GEF activity is unclear. The indirect pathway is more complex and involves a positive feedback component, since the Rabex-5/Rabaptin-5 complex targets to the membrane via binding to the product (Rab5-GTP) to produce more product molecules. In this case, the Rabex-5/Rabaptin-5/Rab5-GTP tripartite complexes act on neighboring substrate molecules (Rab5-GDP) on the membrane to convert them to Rab5-GTP. In the current study, we conduct quantitative analyses of the relative contributions of the direct and indirect pathways to the Rabex-5-mediated Rab5 activation in cultured cells. We develop a mathematical model, and conduct experiments that employ Rabex-5 variants deficient in either direct or indirect pathway to determine the kinetics of each pathway independently.

## Results

### Mathematical Model and “Delayed Onset” of a Rabex-5/Rabaptin-5-Mediated Positive Feedback Loop

**Figure 1:**
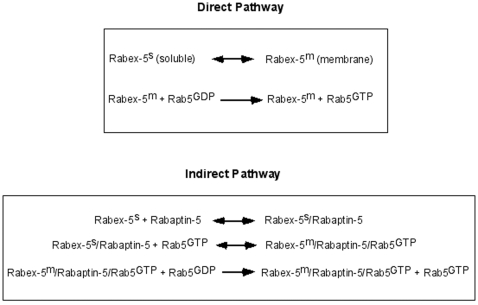
Direct and indirect pathways in Rabex-5-mediated Rab5 activation. Rabex-5 targets to the early endosomal membrane in two parallel pathways: direct targeting via the EET domain and indirect targeting via forming complex with Rabaptin-5 that binds to Rab5-GTP. The reaction scheme of the direct pathway contains two steps: newly synthesized cytosolic Rabex-5 binds to the membrane, and the membrane-bound Rabex-5 can then act on the membrane-bound substrate Rab5-GDP. The reaction scheme of the indirect pathway contains three steps: cytosolic Rabex-5 binds to Rabaptin-5 in the cytoplasm, the Rabex-5/Rabaptin-5 complex remains soluble in the cytoplasm until its level and/or Rab5-GTP level reach a threshold and the complex is recruited to the membrane by binding to Rab5-GTP, and the membrane-bound Rabex-5 in turn converts more Rab5-GDP to Rab5-GTP. The superscripts “s” and “m” denote soluble and membrane-bound forms of Rabex-5 in the cell.

The reaction schemes in the direct and indirect Rab5 activation pathways by Rabex-5 can be described as follows. In the direct pathway, newly made Rabex-5 targets to early endosomes and becomes membrane-bound, then the membrane-bound form of Rabex-5 catalyzes the nucleotide exchange reaction to convert Rab5-GDP to Rab5-GTP (Figure 1). The membrane targeting step is critical for the subsequent interaction between Rabex-5 and Rab5 on the endosomal membrane and we have shown that there is little direct interaction between soluble/cytosolic Rabex-5 and membrane-bound Rab5 in the cell [9]. In the indirect pathway, Rabex-5 associates with Rabaptin-5 in the cytosol (Figure 1). This Rabex-5/Rabaptin-5 complex remains cytosolic until it binds to endosomal Rab5-GTP via Rabaptin-5 and is recruited to the membrane. This tripartite complex (Rabex-5/Rabaptin-5/Rab5-GTP) further activates neighboring Rab5 molecules and converts more Rab5-GDP to Rab5-GTP, which creates a positive feedback loop (Figure 1).

A comprehensive computational model has been developed for a Rho GTPase (cdc42) recently by Goryachev and Pokhilko [14], which provides a general framework for GTPase-regulated processes. The Rab5 GTPase system is unique in the sense that it contains a positive feedback component. To develop a specific mathematical model for Rab5 activation, the reaction scheme is simplified based on experimental observations. For example, we only consider membrane-bound Rab5 because Rab5 is mostly associated with the membrane at steady state in the cells studied here. Newly synthesized Rab5 rapidly associates with the endosomal membrane via its C-terminal prenyl group. In addition, the kinetic constants in the cell are yet to be quantified, which is necessary for more detailed computational analysis. Nonetheless, our mathematical model reveals a new concept of “delayed response” in Rab5 activation and provides a guide for the experiments described below. The data shed light on the relationship of the kinetic parameters.

Define *z* as the concentration of activated, GTP-bound Rab5 in the cell, then the GDP-bound Rab5 concentration is (z_tot_-*z*), where z_tot_ is the total concentration of Rab5 in a cell. The rate of Rab5 activation, i.e., Rab5-GTP production, can be expressed as 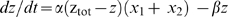
(1)


On the right side of this equation, the first term is Rab5-GTP production catalyzed by total Rabex-5 via both direct and indirect pathways, while the second term reflects GTP hydrolysis catalyzed by Rab5 GAP. The parameters α and β are the rate constants of enzyme reactions catalyzed by the GEF and GAP, respectively. The second-order rate constant α is defined as the rate constant per unit concentration of Rabex-5, while the first-order rate constant β contains the GAP concentration in a cell. *x_1_* is the amount of membrane-bound Rabex-5 via the direct pathway, while *x_2_* is the amount of Rabex-5/Rabaptin-5/Rab5-GTP tripartite complex via the indirect pathway. The key in the mathematical modeling is to develop quantitative descriptions for *x_1_* and *x_2_*.

Define *x_0_* as the amount of cytosolic Rabex-5, which can form complexes with Rabaptin-5, then ***x_1_*** **= σ** ***x_0_***, where σ is the equilibrium constant of the direct membrane targeting pathway (**Figure 1**). We assume rapid equilibrium for the direct membrane targeting of Rabex-5. In the indirect pathway via Rabaptin-5, *x_2_* satisfies 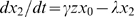
(2)where *γ* and *λ* are the rate constants for the formation and dissociation of the Rabex-5/Rabaptin-5/Rab5-GTP tripartite complex. *γ* contains Rabaptin-5 concentration in the cytosol. Finally total Rabex-5 concentration in a cell can be expressed as 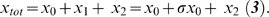 This equation can be reorganized as follows.
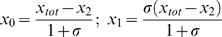
(4)


Bringing these into equations *(2)* and *(1)*, respectively, we have
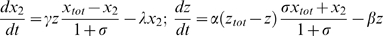
(5)


These are the mathematical descriptions of the kinetics of Rabex-5-mediated Rab5 activation, which includes both direct and indirect pathways.

Based on the above equations, we can titrate Rab5 activation with respect to Rabex-5 activity, i.e., plot *z* (concentration of GTP-bound Rab5) as a function of *x_tot_* (total cellular Rabex-5 concentration). First, we consider a situation if there is no indirect pathway, i.e., *x_2_* = 0, then equation ***(5*** ***)*** at steady state can be simplified as
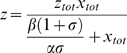
(6)


This reflects that Rabex-5 directly targets to the membrane and activates Rab5, with a kinetics similar to the classical Michaelis-Menten enzyme kinetics, i.e., the response curve is a hyperbola. The slope of the response curve has a Hill's coefficient of 1 with the midpoint for the cellular Rabex-5 concentration, *x_tot_*, being at
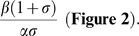



**Figure 2:**
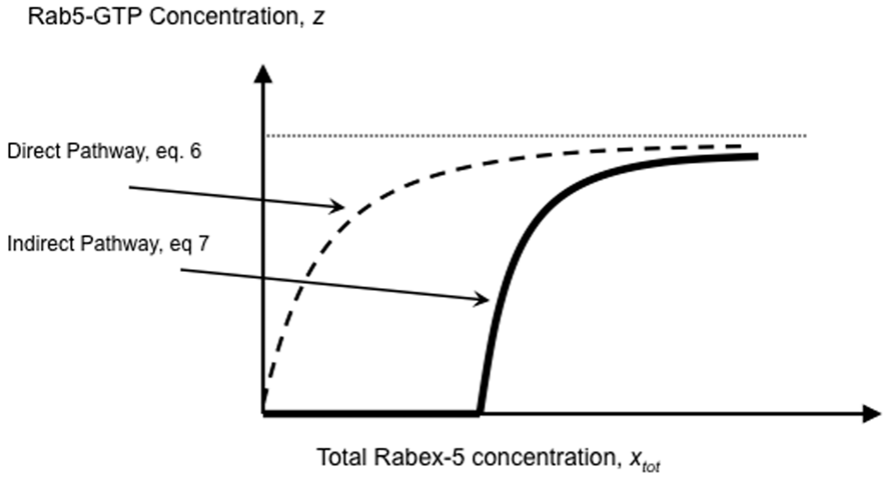
Mathematical model of Rab5 activation via direct and indirect pathways. Rab5 activity is plotted against Rabex-5 concentration in the cell, when there is only direct or indirect pathway as indicated. The response curves are plotted according to the mathematical model (eq. 6 and eq. 7) described in the text, with rate constants arbitrarily assigned as: α = 1, β = 1, γ = 1, λ = 100, and σ = 0.1.

Second, we consider another situation if there is no direct pathway, i.e., **σ** = 0, then solving equations *(5* *)* yields
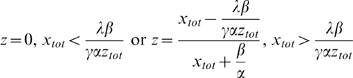
(7)


This response curve via the indirect pathway is completely different from the one via the direct pathway (**Figure 2**). It has a more abrupt or sensitive response with a Hill's coefficient of
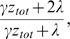
which is greater than 1 and can reach 2 if *λ γ*≫*z_tot_*, i.e., when cellular Rab5 concentration is below the affinity between Rab5-GTP and Rabex-5/Rabaptin-5, with the given Rabaptin-5 concentration contained in *γ*. Here the Hill coefficient concept is generalized to reflect the sensitivity of a transition, i.e., the increase of Rab5-GTP level per percent change of Rabex-5 concentration at the midpoint. It's not simply the slope at the midpoint of a curve, but the slope normalized by the location of the midpoint. The farther away the midpoint is from the origin, the more sensitive the transition is. For example, two identical slopes with midpoints at 10 and 100 would have a 10-fold difference in sensitivity, with the latter more sensitive.

An important characteristic of the curve is the delayed onset of the response, which increases the sensitivity of the transition. A close inspection of the entire curve from the origin (*x_tot_ < γβ/γαz_tot_* plus *x_tot_ > λβ/γαz_tot_*) shows a sigmoidal shape, even though the initial phase is essentially zero before the onset. At low Rabex-5 concentrations, there is no Rab5 activation. When Rabex-5 concentration increases to a threshold level, which is determined by the indicated rate constants, there is a sudden increase of Rab5 activation, due to the positive feedback loop.

The sigmoidal activation of Rab5 by the positive feedback loop in the indirect pathway is further supported by a detailed steady state analysis of the mathematical model (Supplemental Materials S1, Figures S3 and S4). Simulation analysis of the model indicates that the activation curve becomes increasingly sigmoidal with decreasing **σ** (Figure S4). The sigmoidal activation is an intermediate scenario between standard hyperbolic activation and bistability. The system does not exhibit bistability with all possible parameters analyzed (Supplemental Materials S1), because the positive feedback is not sufficiently robust. One of the two steady states is always unstable. The branching is transcritical rather than a saddle-node bifurcation that is usually necessary for bistability. The branching behavior supports our conclusion of the “delayed onset” of Rab5 activation in the indirect pathway.

### Onset of the Positive Feedback Loop Dependent on Rab5 Level

The mathematical model suggested that the Rabex-5 concentration necessary for the onset of the positive feedback loop is related to the ratio of *λβ*vs. *γαz_tot_* and prompted us to determine such threshold Rabex-5 concentration in the cell to gain insight into the relationship of these important rate parameters in Rab5 activation.

**Figure 3:**
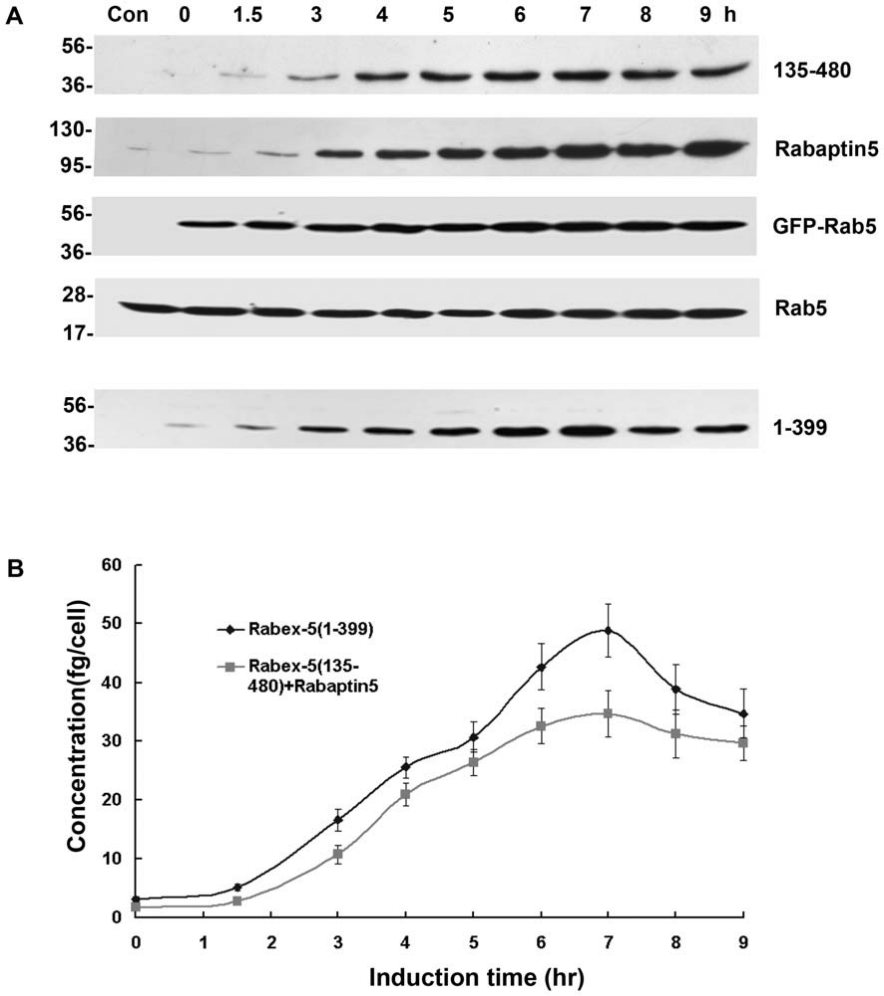
Inducible expression of Rabex-5 constructs and Rabaptin-5 in BHK cells. **A.** BHK cells were transfected with pBI/myc-Rabex-5(135–480)/Rabaptin-5 or pBI/myc-Rabex-5(1–399), pTet-Off, and pcDNA3/GFP-Rab5 (3∶3∶1) and incubated at 37°C for 15 hours in the presence of Dox. Upon removal of Dox, myc-Rabex-5(135–480)/Rabaptin-5 or myc-Rabex-(1–399) expression was induced for the indicated times. Shown are immunoblots of the cell lysates with anti-myc, anti-Rabaptin-5, and anti-Rab5 antibodies. Control cells (Con) were transfected with the empty pBI vector. Endogenous Rab5 serves a loading control. Molecular mass standards (in kDa) are indicated on the left side of the panel. **B.** Shown is the quantification of myc-Rabex-5(135–480) expression from the immunoblot in A by densitometry. The graph shows inducible myc-Rabex-5(135–480) or myc-Rabex-(1–399) expression over the indicated time course, with intracellular protein concentrations calculated based on the standard curve described in the Materials and Methods and error bars indicating SEM from three independent immunoblot experiments.

We followed the kinetics of Rab5 activation by a Rabex-5 truncation mutant [Rabex-5(135–480)] that lacks the EET domain and can only activate Rab5 via the indirect pathway upon association with Rabaptin-5 [9]. In BHK cells with early endosomes labeled by GFP-Rab5, Rabex-5(135–480) was co-expressed with Rabaptin-5 in a single bi-directional expression vector in a Tet-Off system, which allowed suppression and synchronization of protein expression in the presence of Dox (1 µg/ml). Rabex-5(135–480) contained a Myc-epitope at the N-terminus and its expression was confirmed by immunoblot analysis with an anti-Myc monoclonal antibody, while the Rabaptin-5 expression was determined with an anti-Rabaptin-5 antibody (Figure 3A). A small amount of Rabex-5(135–480) was consistently detected even in the presence of Dox (Figure 3A), due to the leakiness of the Tet-Off system. Upon Dox removal, the Rabex-5(135–480) level increased by approximately an order of magnitude within 7 hours when it plateaued and reached a steady state (Figure 3A and 3B). Rabaptin-5 expression from the same plasmid exhibited a similar kinetics, but reached a higher level and increased by more than an order of magnitude over the Dox-suppressed background level in 9 hours (Figure 3A). GFP-Rab5 level in these cells served as an internal control, which was expressed via the pcDNA3 vector and was not regulated by Dox. The GFP-Rab5 expression was allowed to reach steady state, which was approximately twice the amount as endogenous Rab5 (Figure 3A and supplemental Figure S2), before the induction of Rabex-5(135–480)/Rabaptin-5 expression by Dox removal.

In addition to co-expression with Rabaptin-5, Myc-Rabex-5(135–480) was also expressed by itself, with the same inducible expression kinetics, so was the full-length Myc-Rabex-5 (supplemental Figure S1). Furthermore, we expressed Myc-tagged Rabex-5(1–399) in this Tet-Off system for comparison (Figure 3A). The Rabex-5(1–399) construct lacks the Rabaptin-5-binding domain (residues 401–480) but contains the EET domain (residues 81–230) for direct targeting to early endosomes [9]. Upon induction by Dox removal, Rabex-5(1–399) showed the same expression kinetics as Rabex-5(135–480) and the expression increased over time until 7 hours post-induction when it plateaued (Figure 3A and 3B). However, Rabex-5(1–399) appeared less stable than Rabex-5(135–480) and the steady state level decreased after 7 hours (Figure 3A and 3B), possibly due to the presence of their N-terminal ubiquitin-binding domain [12].

**Figure 4:**
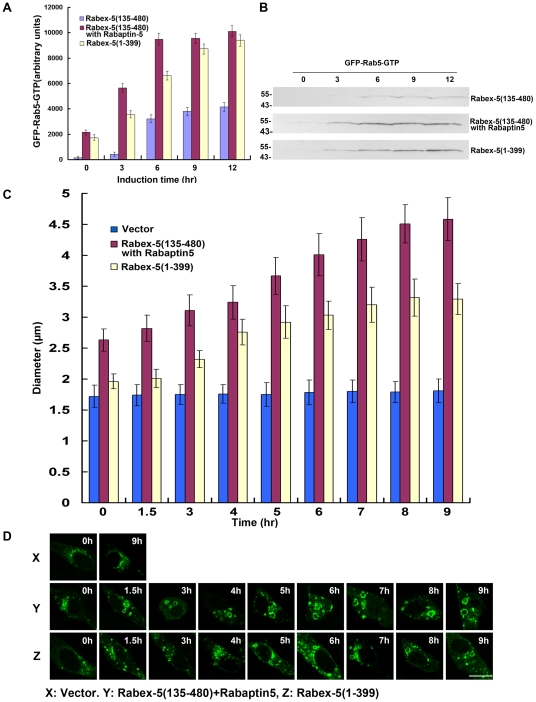
Kinetics of Rabex-5(135–480)/Rabaptin-5 and Rabex-5(1–399)-mediated Rab5 activation in BHK cells with ectopic expression of GFP-Rab5. **A.** GST pull-down assay showing increased levels of GTP-bound GFP-Rab5 over the time course of inducible expression of the indicated Rabex-5 proteins (see Figure 3). GTP-bound GFP-Rab5 in each cell lysate was detected by its binding to GST-R5BD, followed by immunoblot analysis with an anti-Rab5 mAb and quantification by densitometry. Endogenous Rab5-GTP level was too low to be detected with the same amount of lysates. The graph shows the quantification of GTP-bound GFP-Rab5 in each cell lysate, and error bars represent SEM of three independent experiments. Representative immunoblots from one of the experiments are shown in **B.** Molecular mass standards (in kDa) are indicated on the left side of each panel. **C.** Confocal fluorescence microscopy analysis of the size increase of GFP-Rab5-labeled early endosomes over the time course of inducible expression of the indicated Rabex-5 proteins. The graph quantifies the maximal size of early endosomes in cells expressing Rabex-5(1–399) or Rabex-5(135–480) with Rabaptin-5, as indicated. In control cells transfected with the empty vector, the size of endogenous endosomes did not change over time and was slightly smaller than that in cells expressing Rabex-5(1–399) at 0 h (see panel D). The 0 h value of control cells is shown in the graph to serve as a background control. The diameters of 90 largest GFP-Rab5-labeled endosomes in 30 cells were measured in each case and the graph shows the mean and calculated SEM. Representative confocal fluorescence microscopy images of the GFP-Rab5-labeled early endosomes used in the quantification are shown in **D.** X indicates control cells transfected with the empty pBI vector; Y indicates cells expressing Rabex-5(135–480) and Rabaptin-5; Z indicates cells expressing Rabex-5(1–399). Bar = 16 µm.

Next we monitored Rab5 activation in the cell with increasing levels of Rabex-5(135–480)/Rabaptin-5 as well as the other Rabex-5 constructs by determining intracellular Rab5-GTP level with pull-down assays and by determining the maximum size of GFP-Rab5-labeled early endosomes, which correlates with Rab5-GTP level, via confocal fluorescence microscopy (Figure 4). With increased expression of Rabex-5(135–480) and Rabaptin-5, thus more Rabex-5(135–480)/Rabaptin-5 complexes in the cell, there was a correlated increase in Rab5 activity, as evidenced by increased levels of GFP-Rab5-GTP (Figure 4A and 4B) and by the enlargement of GFP-Rab5-labeled early endosomes (Figure 4C and 4D). Importantly, the level of GFP-Rab5-GTP positively correlated with the enlargement of GFP-Rab5-labeled early endosomes (Figure 4). In both assays, a significant increase in Rab5 activity was observed in the presence of Dox (Figure 4), a condition that suppressed Rabex-5(135–480) expression by an order of magnitude and only showed a low residual level of the protein in the cell (Figure 3). Indeed, the Rabex-5(135–480) level in this case was similar to the endogenous Rabex-5, as determined by comparison of the level of Myc-tagged full-length Rabex-5 expressed via the same Tef-Off vector in the presence of Dox, with that of endogenous Rabex-5 detected by an anti-Rabex-5 antibody (supplemental Figure S1). This low level of Rabex-5(135–480), with Rabaptin-5, was able to increase Rab5 activity, suggesting that endogenous Rabex-5/Rabaptin-5 level was already near or above the threshold level for the onset of the positive feedback loop in Rab5 activation and there was no delayed phase in these cells, given that Rab5 was overexpressed by 2-fold (in the form of GFP-Rab5). The Rabex-5(135–480) activity was dependent on co-expressed Rabaptin-5 and Rabex-5(135–480) expressed alone showed little activity (Figure 4A and 4B), indicating that endogenous Rabaptin-5 was limiting for formation of new complexes.

To determine the kinetics of Rab5 activation mediated by the direct pathway, i.e., direct membrane targeting of Rabex-5 and activation of Rab5, we took advantage of the Rabex-5 truncation mutant [Rabex-5(1–399)] that lacks the Rabaptin-5-binding domain and can only targets to the early endosomes via the direct pathway mediated by the EET domain [9]. This mutant was expressed in BHK cells in the same fashion as Rabex-5(135–480) and the effect on Rab5 activation was determined as described above. With increasing Rabex-5(1–399) expression during the time course (Figure 3A and 3B), there was correlated increase in the level of GFP-Rab5-GTP (Figure 4A and 4B) and in the size of early endosomes (Figure 4C and 4D), which reflected increased Rab5 activity in the cell. However, the kinetics of Rabex-5(1–399)-mediated Rab5 activation was slower than that of Rabex-5(135–480)/Rabaptin-5-mediated Rab5 activation (Figure 4), consistent with our mathematical model that the positive feedback loop contained in the latter (indirect) pathway promotes a faster kinetics. Expression of full-length Rabex-5 alone showed the same slow activation kinetics as Rabex-5(1–399) (data not shown), confirming that Rabaptin-5 is limiting in the cell and overexpressed Rabex-5 mostly targets to the early endosomes and activates Rab5 via the direct pathway.

**Figure 5:**
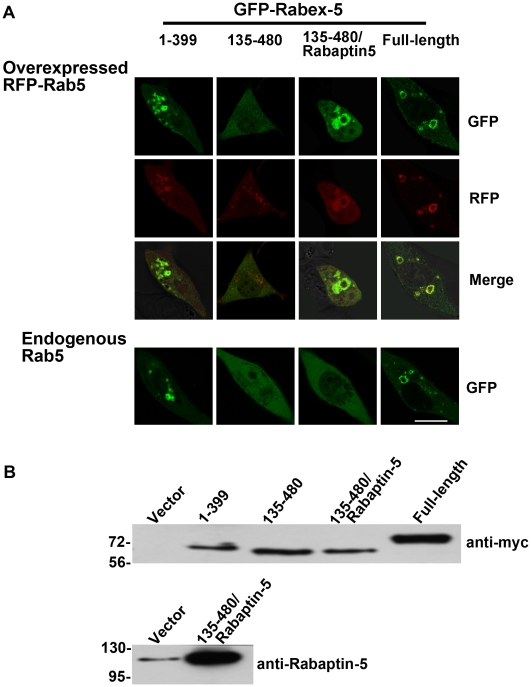
Only the direct pathway allows Rabex-5 to target to early endosomes and activate Rab5 in BHK cells with endogenous level of Rab5. **A.** Confocal fluorescence microscopy images of BHK cells expressing the indicated GFP-Rabex-5 proteins with or without co-expression with RFP-Rab5. The cells were transfected with the indicated constructs and processed for microscopy 15 hours after the transfection. Bar = 16 µm. **B.** Immunoblots showing the expression of the indicated GFP-Rabex-5 constructs and Rabaptin-5. The GFP-Rabex-5 constructs contain a myc-epitope downstream of GFP and was probed with the anti-myc antibody (top panel), while Rabaptin-5 expression was identified with the anti-Rabaptin-5 antibody (bottom panel). Molecular mass standards (in kDa) are indicated on the left side of each panel.

The data suggest that endogenous levels of Rabex-5/Rabaptin-5 are sufficient for the onset of the positive feedback loop in Rab5 activation, given that the cells overexpress Rab5 by 2-fold (in the form of GFP-Rab5) (Figure 3A and supplemental Figure S2). We then determined kinetics of endogenous Rab5 activation by Rabex-5(135–480)/Rabaptin-5 and the other Rabex-5 constructs. In this case, the Rabex-5 constructs were tagged with GFP at the N-terminus and their localization and activity were confirmed by a control experiment in which each GFP-Rabex-5 construct was co-expressed with RFP-Rab5 in the cell, followed by confocal fluorescence microscopy (Figure 5A). GFP-Rabex-5 and GFP-Rabex-5(1–399) correctly targeted to RFP-Rab5-labeled early endosomes and enhanced Rab5 activity, as evidenced by the enlargement of these early endosomes (Figure 5A). GFP-Rabex-5(135–480) alone showed diffused cytoplasmic staining, but GFP-Rabex-5(135–480)/Rabaptin-5 co-expression lead to co-localization of GFP-Rabex-5(135–480) with RFP-Rab5 on the early endosomes and enlargement of these early endosomes (Figure 5A), similar to the results obtained with Myc-Rabex-5(135–480)/Rabaptin-5 (Figure 4).

Without ectopic expression of RFP-Rab5 in the cell, however, GFP-Rabex-5(135–480)/Rabaptin-5 expression alone was unable to target GFP-Rabex-5 (135–480) to early endosomes, instead it exhibited diffused cytoplasmic staining (Figure 5). In contrast, both GFP-Rabex-5 and GFP-Rabex-5(1–399) showed punctate staining pattern, suggesting that they targeted to early endosomes, and importantly increased Rab5 activity leading to enlarged endosomes (Figure 5). Both constructs activate Rab5 via the direct pathway independent of Rabaptin-5, even though the full-length Rabex-5 contains Rabaptin-5 binding domain but endogenous Rabaptin-5 is limiting and unavailable for new complex formation (Figure 4) [9]. The data indicate that at endogenous level of Rab5, only the direct pathway allows Rabex-5 to target to early endosomes and activate Rab5, while the indirect pathway, which relies on Rab5-GTP for recruitment of Rabex-5/Rabaptin-5 complexes to the endosomes, cannot function effectively. We interpret this observation as a result of less than threshold level of Rab5-GTP on the endosomes. In this regard, the ectopic expression of Rabex-5(135–480)/Rabaptin-5 is apparently insufficient to compensate for the 50% decrease in the Rab5 level, indicating that the onset of the Rabex-5/Rabaptin-5-mediated positive feedback activation of Rab5 is highly sensitive to the Rab5 concentration in the cell, as predicted by the model (equation 7).

**Figure 6:**
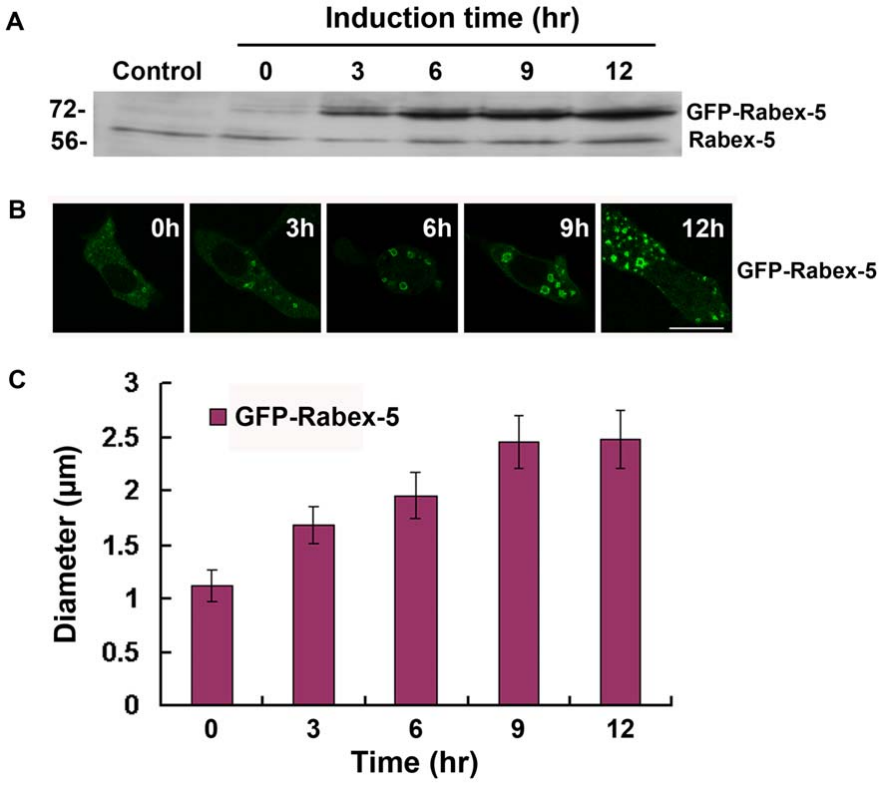
Kinetics of GFP-Rabex-5-mediated Rab5 activation in BHK cells with endogenous level of Rab5. **A.** Immunoblot showing inducible expression of GFP-Rabex-5. BHK cell monolayers were transfected either with the empty pBI vector (control) or with pBI/GFP-Rabex-5 and pTet-Off, then incubated at 37°C for 15 hours in the presence of Dox. Upon Dox removal to induce GFP-Rabex-5 expression, cell lysates were prepared at the indicated times for immunoblot analysis with the anti-Rabex-5 antibody. Endogenous Rabex-5 in the same lysates serves an internal loading control, as indicated. Molecular mass standards (in kDa) are indicated on the left side of the panel. **B.** Confocal fluorescence microscopy showing the size increase of GFP-Rabex-5-labeled early endosomes over the time course of inducible expression of the protein as indicated. Bar = 16 µm. **C.** The graph quantifies the maximum size of GFP-Rabex-5-labeled early endosomes shown in B. The diameters of 90 largest GFP-Rabex5-labeled endosomes in 30 cells were measured in each case and the graph shows the mean and calculated SEM.

We then focused on the kinetics of GFP-Rabex-5(1–399)- and GFP-Rabex-5-mediated activation of endogenous Rab5. GFP-Rabex-5 showed higher expression level than GFP-Rabex-5(1–399) at steady state (Figure 5B) and allowed us to examine the kinetics of Rab5 activation in a wider range of Rabex-5 concentration in the cell (Figure 6). In the presence of Dox, GFP-Rabex-5 expression was suppressed. However, there was a low background expression of GFP-Rabex-5 that was less than endogenous Rabex-5 level (Figure 6A), due to leakiness of the system. This low level of GFP-Rabex-5 was detected on punctate early endosomes in the cell by confocal fluorescence microscopy (Figure 6B). Upon removal of Dox to induce GFP-Rabex-5 expression, GFP-Rabex-5 level increased over time (Figure 6A), accompanied by increased size of early endosomes reflecting the increasing Rab5 activity in the cell (Figure 6B and 6C).

The data indicate that while the direct pathway is active, the indirect pathway (i.e., Rabex-5/Rabaptin-5-mediated positive feedback loop) is inactive in BHK cells, at endogenous levels of Rab5 and Rabex-5. In other words, the onset of the positive feedback loop is significantly delayed. An order of magnitude of increase in the enzyme [Rabex-5(135–480)] concentration to 32 fg/cell (Figure 3) is still less than the threshold level for the onset of the positive feedback loop. According to our mathematical model (equation 7), the ratio of λβ vs. *γαz_tot_* should be greater than 32 fg/cell in these cells. Note that λ and β are first-order rate constants, while *γ* and *α* are second-order rate constants. While the dissociation and associate rates (λ and γ) for Rabex-5/Rabaptin-5 and Rab5-GTP interaction are unknown, the rates for GAP-accelerated GTP hydrolysis by Rab5 (β) and Rabex-5-catalyzed GDP dissociation on Rab5 (α) in the cell can be estimated by *in vitro* biochemical data. The intrinsic GTP hydrolysis rate constant by Rab5 at 37°C is 2×10^−3^ s^−1^ [15]. The rate should be enhanced by at least two orders of magnitude by RabGAP5 [16] and possibly other GAPs in the cell [17]. Thus β is estimated to be 2×10^−1^ s^−1^ per cell. The Rabex-5-catalyzed GDP dissociation rate on Rab5 is 2×10^4^ M^−1^ s^−1^ *in vitro* [8]. Assuming that the value holds true in the cell, the ratio of β and α is determined as 10^−5^ M.

Next we convert 32 fg/cell of Rabex-5(135–480) to molar concentration, which is determined as 10^−5^ M in the cell, taken into consideration of the mean cell volume (∼80 fL) and molecular weight of Rabex-5(135–480) (40 kDa). Bring the values into the model, our data suggest that the ratio of *λ/γz_tot_* should be greater than 1. In BHK cells, endogenous Rab5 concentration (*z_tot_*) is calculated as 6×10^−6^ M (supplemental Figure S2), thus the ratio of *λ/γ* should be greater than 6×10^−6^ M, which reflects the dissociation and association rates (affinity) of Rabex-5/Rabaptin-5/Rab5-GTP complex in BHK cells, given the Rabaptin-5 concentration contained in *γ*.

The equation 7 of the mathematical model predicts that the delayed onset threshold, *λβ/γαz_tot_*, is inversely related to the total Rab5 concentration, *z_tot_*, in the cell. Indeed our data show that overexpression of Rab5 (in the form of GFP-Rab5) in the cell can significantly reduce the Rabex-5 concentration necessary for the onset of the positive feedback loop (Figure 4), i.e., endogenous Rabex-5 concentration (∼3 fg/cell) became sufficient for the onset of positive feedback activation. The trend or change is qualitatively consistent with the model. However, the presence of endogenous Rabex-5, i.e., σ is not zero, prevented more quantitative analysis in BHK cells by the model. In contrast, NF73 cells lacking endogenous Rabex-5 [7] allowed us to determine the ratio of *λ/γ* (see below).

### Delayed Onset of the Positive Feedback Activation of Rab5 in the Absence of the Direct Pathway and Determination of the Affinity (λ/γ) of Rabex-5/Rabaptin-5/Rab5-GTP Complex in NF73 Cells

**Figure 7:**
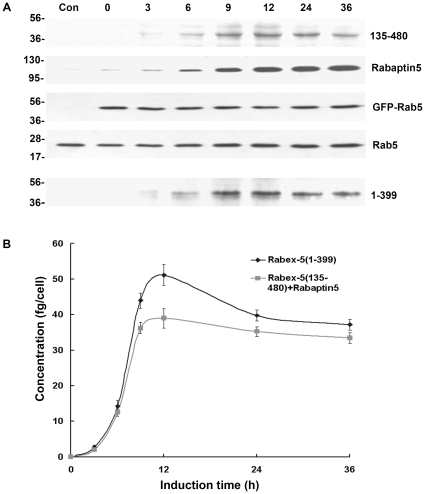
Inducible expression of Rabex-5 constructs and Rabaptin-5 in Rabex-5-deficient mouse embryo fibroblasts. **A.** Cells were transfected with pBI/myc-Rabex-5(135–480)/Rabaptin-5 or pBI/myc-Rabex-5(1–399), pTet-Off, and pcDNA3/GFP-Rab5 (3∶3∶1) and incubated at 37°C for 15 hours in the presence of Dox. Upon removal of Dox, myc-Rabex-5(135–480)/Rabaptin-5 or myc-Rabex-5(1–399) expression was induced for the indicated times. Shown are immunoblots of the cell lysates with anti-myc, anti-Rabaptin-5, and anti-Rab5 antibodies. Control cells were transfected with the empty pBI vector. Endogenous Rab5 in the same lysates serves an internal loading control. Molecular mass standards (in kDa) are indicated on the left side of the panel. **B.** Shown is the quantification of myc-Rabex-5(1–399) or myc-Rabex-5(135–480) expression from the immunoblot in A by densitometry. The graph shows inducible myc-Rabex-5(135–480) or myc-Rabex-(1–399) expression over the indicated time course, with intracellular protein concentrations calculated based on the standard curve described in the Materials and Methods and error bars indicating SEM from three independent immunoblot experiments.

**Figure 8:**
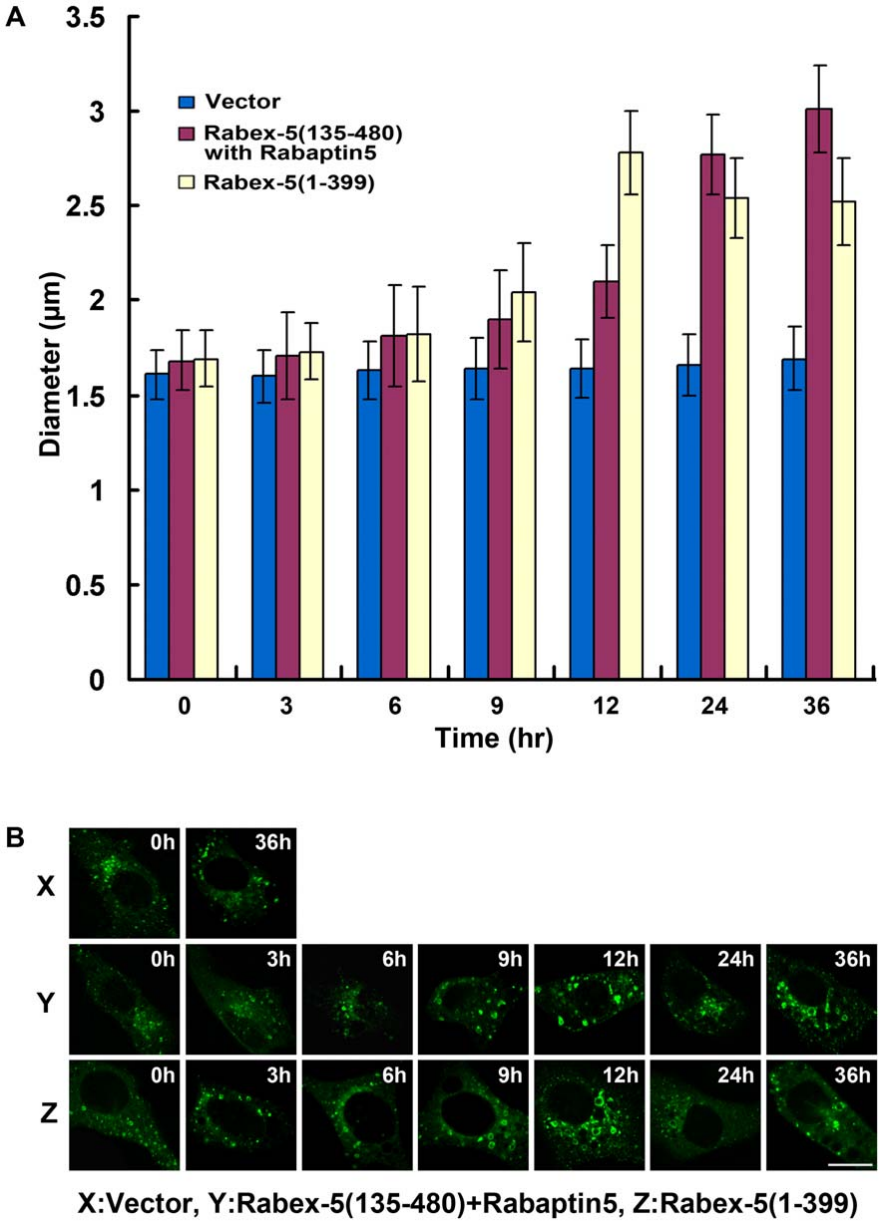
Kinetics of Rabex-5(135–480)/Rabaptin-5 and Rabex-5(1–399)-mediated Rab5 activation in Rabex-5-deficient cells with ectopic expression of GFP-Rab5. **A.** Confocal fluorescence microscopy analysis of the size increase of GFP-Rab5-labeled early endosomes over the time course of inducible expression of the indicated Rabex-5 proteins (see Figure 7). The graph quantifies the maximal size of early endosomes in cells expressing Rabex-5(1–399) or Rabex-5(135–480) with Rabaptin-5, as indicated. In control cells transfected with the empty vector, the size of endogenous endosomes did not change over time and was similar to that in cells expressing Rabex-5(1–399) or Rabex-5(135–480) at 0 h (see panel B). The 0 h value of control cells is shown in the graph to serve as a background control. The diameters of 90 largest GFP-Rab5-labeled endosomes in 30 cells were measured in each case and the graph shows the mean and calculated SEM. Representative confocal fluorescence microscopy images of the GFP-Rab5-labeled early endosomes used in the quantification are shown in **B.** X indicates control cells transfected with the empty pBI vector; Y indicates cells expressing Rabex-5(135–480) and Rabaptin-5; Z indicates cells expressing Rabex-5(1–399). Bar = 16 µm.

The importance of the direct pathway in Rab5 activation became apparent when the kinetics was examined in Rabex-5-deficient NF73 cells, which were mouse embryo fibroblasts (MEF) isolated from Rabex-5 knockout mice [7]. In this case, there was no endogenous Rabex-5 to activate Rab5 and provide a basal level of Rab5-GTP in the cell, via either direct or indirect pathway. To determine the kinetics of the positive feedback loop (i.e., the indirect pathway) in these cells, Rabex-5(135–480) was expressed, either alone or with Rabaptin-5, and there was ectopic expression of GFP-Rab5 for labeling the early endosomes (Figures 7 and 8), as described above for the experiments in BHK cells (Figures 3 and 4). The expression of the Rabex-5 proteins took longer time (12 hours after induction) to reach plateau in these Rabex-5-deficient MEF (Figure 7) than in BHK cells (Figure 3). As a result, it took longer time (6–9 hours after induction) to observe significant increases in Rab5 activity and endosomal size (Figure 8). Expression of Rabex-5(135–480) alone did not show any increase in Rab5 activity and consequently there was no increase in the level of GFP-Rab5-GTP (data now shown), indicating that even though Rabex-5(135–480) may form functional complexes with endogenous Rabaptin-5 for the indirect pathway, the level of any Rabex-5(135–480)/Rabaptin-5 complex formed in this case is likely below the threshold level for the onset of the positive feedback loop and is insufficient to activate Rab5, as predicted by the mathematical model (Figure 2). Indeed when Rabex-5(135–480) was co-expressed with Rabaptin-5, there was corresponding increase in Rab5 activity, as evidenced by the increased size of GFP-Rab5-labeled early endosomes (Figure 8). However, the kinetics of this positive feedback loop-mediated Rab5 activation was delayed relative to Rabex-5(1–399)-mediated Rab5 activation, via the direct pathway (Figure 8), in contrast to the immediate onset of the positive feedback loop in BHK cells under the same condition of GFP-Rab5 overexpression (Figure 4). Thus in Rabex-5-deficient cells with no directly targeted endogenous Rabex-5 on the early endosomes to provide Rab5-GTP (i.e., σ = 0), the Rabex-5(135–480)/Rabaptin-5-mediated Rab5 activation is delayed, even when there is Rab5 overexpression.

The data are consistent with the model. Furthermore, the data suggest that the onset of positive feedback activation of Rab5 starts between 6 and 9 hours after inducible expression of Rabex-5(135–480)/Rabaptin-5 (Figure 8). The Rabex-5(135–480) concentration is approximately 12 fg/cell at 6-hour post induction (Figure 7), which is used to estimate the ratio of *λ* vs. *γ* according to the model (equation 7), i.e., 12 fg/cell should equal the ratio of *λβ* vs. *γαz_tot._* Taken into consideration of the ratio of β and α discussed above (10^−5^ M) as well as the total Rab5 concentration (endogenous Rab5 and GFP-Rab5), z_tot_, in the cell (2.5×10^−5^ M) (Supplemental Figure S2), the ratio of *λ/γ* is calculated as 10^−5^ M, which reflects the affinity of the Rabex-5/Rabaptin-5/Rab5-GTP tripartite complex in the cell. A caveat is that the estimated 100-fold GAP-mediated enhancement in β is at the low end of the 2 to 5 orders of magnitude enhancement among GAP-accelerated rates of other Ras-related GTPases [17]. Should the Rab5 GAP(s) turns out to be more powerful, the ratio of *λ/γ* could be reduced accordingly. Thus with the guidance of the mathematical model, we are able to estimate the kinetic parameters of Rabex-5/Rabaptin-5/Rab5-GTP interaction in the cell for the first time.

## Discussion

Rabex-5 functions as a GEF for Rab5 activation in the cell and plays a critical role in regulation of early endosome fusion and endocytosis [1], [10]. Rabex-5 knockout mice develop severe skin inflammation and die early, indicating a non-redundant and essential function *in vivo* [6]. Although there are other Vps9 domain-containing Rab5 GEFs, such as the RIN proteins, they are subject to temporal regulation and become active only upon growth factor-mediated activation and recruitment to early endosomes during signal transduction processes [18]. In the current study, we have examined the impact of concentration changes of Rabex-5 on Rab5 activation and early endosome dynamics in the cell, via mathematical modeling and kinetic studies of intracellular levels of Rab5-GTP and enlargement of early endosomes. We have employed a tetracycline-regulated expression system to synchronize the expression of two Rabex-5 constructs [Rabex-5(1–399) and Rabex-5(135–480)] that activate Rab5 via direct and indirect pathways, respectively. Our data demonstrate that Rab5 activity and early endosomal dynamics is highly sensitive to alterations in Rabex-5 concentration in the cell.

Rabex-5 can activate Rab5 on early endosomes via direct and indirect pathways [9]. The direct pathway involves direct membrane targeting of Rabex-5 to early endosomes, while the indirect pathway requires Rabex-5 to associate with Rabaptin-5 in the cytosol and the complex then targets to early endosomes via Rabaptin-5 binding to Rab5-GTP. There is little free Rabex-5 in the cytosol because of rapid association with the membrane or Rabaptin-5. Thus there are two major pools of Rabex-5 molecules in the cell at steady state: a membrane-associated pool and a cytosolic pool of Rabex-5/Rabaptin-5 complexes. The former activates Rab5 and produces a basal level of Rab5-GTP on the membrane, which in turn recruits the latter to the membrane for further Rab5 activation, creating a positive feedback loop. Our mathematical model suggests a delay of onset for the positive feedback activation, depending on the levels of Rabex-5/Rabaptin-5 complexes in the cytosol and Rab5-GTP on the membrane. Indeed, endogenous Rab5 appears below the threshold level for the onset of positive feedback activation in BHK cells, since increased expression of Rabex-5(135–480) by an order of magnitude, together with Rabaptin-5, shows no detectable enlargement of early endosomes in the cell, suggesting no increase in Rab5 activity. However, if Rab5 level is increased by 2-fold as in the case of GFP-Rab5 expression, a small increase in Rabex-5(135–480) and Rabaptin-5 leads to easily detectable increase in Rab5 activity and enlargement of early endosomes. This increase is due to the onset of the positive feedback loop, because Rabex-5(135–480) can only use the indirect pathway to associate with early endosomes and activate Rab5. Furthermore, this positive feedback activation shows a faster kinetics than Rabex-5(1–399)-mediated Rab5 activation that functions via the direct pathway.

The Rabex-5-deficient NF73 cells provide a clean background for kinetic dissection of the direct and indirect pathways independently. In these cells, the delayed onset of the positive feedback loop is more obvious and can be observed even when there is over-expression of GFP-Rab5. Upon expressing Rabex-5(135–480) and forming complex with Rabaptin-5, the indirect pathway is restored but the direct pathway remains missing, i.e., σ = 0. Without the direct pathway (endogenous Rabex-5) to provide Rab5-GTP, however, there is extra “burden” on the indirect pathway and requires a higher level of the enzyme, i.e., the Rabex-5(135–480)/Rabaptin-5 complex, to reach the threshold for the onset of the positive feedback loop. Indeed, the Rabex-5(135–480)/Rabaptin-5 –mediated activation and enlargement of early endosomes is significantly delayed relative to the Rabex-5(1–399)-mediated activation, via the direct pathway, in these cells. In contrast, in normal BHK cells where there is endogenous Rabex-5, the over-expression of GFP-Rab5 readily diminishes the delay of Rabex-5(135–480)/Rabaptin-5-mediated positive feedback activation. Thus the biological significance of the direct pathway lies in providing a relatively high basal level of Rab5-GTP to allow the onset of the indirect pathway at relatively low concentrations of the Rabex-5/Rabaptin-5 complex in the cell. The partition of Rabex-5 to indirect or direct pathway depends on the relative concentrations of cytosolic Rabaptin-5 vs. the membrane-binding site, which is recently reported to be another early endosomal Rab, Rab22 [19]. Endogenous ratio of Rabaptin-5 and Rab22 varies among cell types, suggesting functional preference for different endocytic rates by different cell types. The indirect pathway with its positive feedback loop offers great sensitivity to fluctuations in Rabex-5 and Rab5 concentrations in the cell. Thus the collaboration of the direct and indirect pathways balances the GTP hydrolysis rate of Rab5 to produce a steady state level of Rab5-GTP for early endosome fusion and endocytosis.

An important finding of the mathematical model is the relationship between the Rabex-5 concentration for the onset of the positive feedback pathway and the ratio of *λβ* vs. *γαz_tot_*, which are kinetic parameters in Rab5 activation. Our data confirm that increasing Rab5 concentration in the cell (*z_tot_*) reduces the threshold Rabex-5 concentration for the positive feedback pathway. In addition, with expression of Rabex-5(135–480) that can only activate Rab5 via the indirect positive feedback pathway in NF73 cells, which contains no endogenous Rabex-5 and thus lacks any direct pathway-mediated Rab5 activation (σ = 0), we have been able to identify the threshold Rabex-5(135–480) concentration for the positive feedback pathway and determine the ratio of *λ* and *γ* as 10^−5^ M, which reflects the affinity of the Rabex-5/Rabaptin-5/Rab5-GTP complex in the cell.

## Materials and Methods

### Mammalian Cell Cultures and Transfection

Rabex-5-deficient mouse embryo fibroblasts (MEF) [7] were kindly provided by Dr. Galli's lab at Stanford University and cell monolayers were grown in 35-mm culture dishes with 3 ml of DMEM containing 10% fetal bovine serum (Invitrogen). BHK cells were cultured as described previously [9]. Cells were transfected with the plasmid constructs capable of expressing Rabex-5, Rabaptin-5, or Rab5 proteins as indicated via Fugene HD-mediated procedure (Roche Applied Science) and incubated at 37°C in a tissue culture incubator with 5% CO_2_. The expression plasmids used included pcDNA3.1 (Invitrogen) and pBI (Clontech). The pBI vector requires co-transfection with pTet-Off and can express two cloned proteins simultaneously and in a tetracycline or doxycycline (Dox)-regulated manner. Protein expression was confirmed by immunoblot analysis and intracellular localization and endosomal morphology were determined by confocal fluorescence microscopy (see below).

### Immunoblot Analysis

Cells were lysed in 1% SDS (200 µl per dish) and the lysates were sheared to reduce the stickiness by passing through a 26G needle 5 times with a 1-ml syringe, followed by SDS-PAGE and immunoblot assay with ECL reagents (GE Healthcare). The primary antibodies used in these assays included anti-myc monoclonal antibody (Sigma), anti-Rabex-5 antibody (BD Biosciecnes), and anti-Rabaptin-5 antibody (BD Biosciences) as indicated. The immunoblot results were quantified by densitometry with a Densitometer SI (Molecular Dynamics).

### Protein Quantification

Myc-Rabex-5(135–480), Myc-Rabex-5(1–399), and Rab5 were cloned in the pGEX-4T-2 for expression as GST fusion proteins, which were purified as described previously [9]. The GST fusion proteins were bound on glutathione-Sepharose resin and were cleaved by thrombin overnight at room temperature, to release free Myc-Rabex-5(135–480), Myc-Rabex-5(1–399), and Rab5, which were analyzed by 12% SDS-PAGE, and visualized by Coomassie brilliant blue staining. The protein concentrations were determined by the Bio-Rad Protein Assay kit.

The purified Myc-Rabex-5(135–480), Myc-Rabex-5(1–399), and Rab5 were diluted into a series of concentrations (1000 ng, 500 ng, 250 ng, 200 ng, 150 ng, 100 ng, 50 ng, 25 ng, 10 ng/ml) as protein standards, which were subjected to the same immunoblot analysis with the anti-Myc or anti-Rab5 mAb, as the cell lysates containing Myc-Rabex-5(135–480), Myc-Rabex-5(1–399), or Rab5/GFP-Rab5. Signal intensity of protein standards was determined by using Fluorchem Imager with object average background correction applied. Data were exported to Microsoft Excel to generate the standard curve. The background corrected density of each band correlated to the amount of purified Myc-Rabex-5(135–480), Myc-Rabex-5(1–399), or Rab5 protein. The concentrations of Myc-Rabex-5(135–480), Myc-Rabex-5(1–399), endogenous Rab5, and GFP-Rab5 in cell lysates were then quantified with the standard curve and the image analysis program. Protein concentrations were then corrected by cell numbers in the lysates and transfection efficiency (80% for BHK cells and 20% for NF73 cells). Error bars indicate SEM in three experiments.

### Confocal Fluorescence Microscopy

We used a Leica confocal laser scanning microscope with Ar-488 and Kr-568 laser excitation in the Flow and Image Cytometry lab on campus. BHK-21 and Rabex-5-deficient MEF cells were grown on coverslips and transfected with pBI and pcDNA3 constructs expressing various Rabex-5, Rabaptin-5, and Rab5 proteins as indicated. The pBI constructs expressing Myc-tagged Rabex-5 and/or Rabaptin-5 proteins were co-transfected with the pcDNA3 construct expressing GFP-Rab5, while the pBI constructs expressing GFP-tagged Rabex-5 proteins were transfected alone. Cells were incubated at 37°C in the presence of Dox (1 µg/ml) to suppress the pBI construct-mediated expression. After 15 hours when the pcDNA3 construct-mediated expression of GFP-Rab5 reached steady state, Dox was removed by replacing with fresh medium to induce the expression of Rabex-5 and/or Rabaptin-5. At different times as indicated, cells were processed for confocal fluorescence microscopy to determine the morphology of GFP-Rab5- or GFP-Rabex-5-labeled early endosomes. In this case, cells were rinsed three times with PBS and fixed for 20 min with 4% paraformaldehyde (W/V in PBS) at room temperature. The cover slips were then mounted in PBS on glass slides and viewed with the microscope.

### GST Pull-Down Assay

GST-R5BD was produced in *E.coli*, affinity-purified with glutathione-Sepharose 4B resin (GE Healthcare), and used for the pull-down assay as described previously [20]. Briefly, GFP-Rab5 was expressed in BHK cells by transfection of corresponding pcDNA3.1/GFP-Rab5 construct and incubation at 37°C for the indicated times. Cells were lyzed for 5 min in the lysis buffer (200 µl per 35-mm dish), which contained 25 mM HEPES (pH 7.4), 100 mM NaCl, 5 mM MgCl_2_, 0.1% NP40, 10% glycerol, 1 mM DTT and protease inhibitor cocktail (Sigma-Aldrich). Cell lysates were clarified by centrifugation at 10,000 g for 2 min at 4°C and the supernatant was incubated with 20 µl of GST-R5BD bound to the glutathione-Sepharose 4B resin for 10 min at 4°C on a rotating mixer. The resin was subsequently rinsed with the lysis buffer, resuspended in SDS sample buffer, boiled for 3 min, and subjected to SDS-PAGE (15% gel), followed by immunoblot analysis with an anti-Rab5 mAb (BD Biosciences). The results were quantified by densitometry using Densitometer SI (Molecular Dynamics).

## Supporting Information

Materials S1 Steady State Analysis of the Mathematical Model for Delayed Onset of Rab5 Activation by Rabex-5 and Rabaptin-5.
(0.18 MB DOC)

Figure S1 Inducible expression of Rabex-5 constructs in BHK cells. Shown are immunoblots done in parallel with those in Figure 3, indicating the inducible expression of different myc-tagged Rabex-5 constructs without Rabaptin-5. The experiments were the same as that in Figure 3, except pBI/myc-Rabex-5(135–480)/Rabaptin-5 was substituted by pBI/myc-Rabex-5(135–480) and pBI/myc-Rabex-5, respectively, in the transfection as indicated. The expression of each construct was identified by the anti-myc mAb. The full-length myc-Rabex-5 expression was also probed by the anti-Rabex-5 antibody to gauge the level of ectopic expression over endogenous Rabex-5. Molecular mass standards (in kDa) are indicated on the left side of the panel.
(0.43 MB TIF)

Figure S2 Quantification of endogenous Rab5 and GFP-Rab5 in BHK and NF73 cells. A. Shown are endogenous Rab5 and GFP-Rab5 concentrations in BHK cells. The quantification was conducted by immunoblot analysis with an anti-Rab5 mAb (see Figure 3), by comparison with a standard curve generated with known concentrations of recombinant Rab5, as described in Materials and Methods. The values for GFP-Rab5 were corrected by transfection efficiency of BHK cells (80%). In the text, the values of 12 fg/cell (Rab5) and 25 fg/cell (GFP-Rab5) were used to convert to molar concentrations and both were determined to be 6×10^−6^ M considering their difference in molecular weight. B. Shown are endogenous Rab5 and GFP-Rab5 concentrations in NF73 cells. The quantification was conducted as described above (see Figure 7). The values for GFP-Rab5 were corrected by transfection efficiency of NF73 cells (20%). In the text, the values of 25 fg/cell (Rab5) and 40 fg/cell (GFP-Rab5) were used to convert to molar concentrations and both were determined to be 1.2×10^−5^ M considering their difference in molecular weight.
(1.68 MB TIF)

Figure S3 A. Two curves in Eqs. (S3) and (S4), with σ > 0. B. The two curves in Eqs. (S3) and (S4), with σ = 0, have a unstable steady state at the origin and a stable positive steady state. This requires x_tot_ > λβ/(γαz_tot_). C. The two curves in Eqs. (S3) and (S4), with σ = 0, has a negative steady state that requires x_tot_ < λβ/(γαz_tot_). In this case, the zero steady state is stable.
(2.77 MB TIF)

Figure S4 z/z_tot_ as a function of x_tot_ according to Eq. (S10) with the parameters given in Eq. (S12). A. Abscissa in terms of logarithmic x_tot_. B. Abscissa in terms of linear x_tot_. The activation curve is hyperbolic for large σ and sigmoidal for small σ. The delayed onset occurs at x_tot_ = 10.
(0.07 MB TIF)
