# Supplementary material for: Delayed Onset of Positive Feedback Activation of Rab5 by Rabex-5 and Rabaptin-5 in Endocytosis
Source: PLoS One. 2010 Feb 16;5(2):e9226. doi: 10.1371/journal.pone.0009226 (PMC2821916; doi:10.1371/journal.pone.0009226)
Supplement: Materials S1 — Steady State Analysis of the Mathematical Model for Delayed Onset of Rab5 Activation by Rabex-5 and Rabaptin-5. (0.18 MB DOC) [file pone.0009226.s001.doc]

**Steady State Analysis of a Mathematical Model for Signal Activation with Delayed Onset[[1]](#footnote-2)**

Huaiping Zhu^1^, Hong Qian^2^ and Guangpu Li^1^

^1^Department of Biochemistry and Molecular Biology

University of Oklahoma Health Sciences Center

Oklahoma City, OK 73104, USA

^2^Department of Applied Mathematics

University of Washington, Seattle, WA 98195, USA

The mathematical model is represented by the planar system of ordinary differential equations


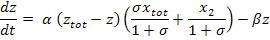

 (S1)


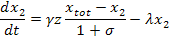

 (S2)

in which *z* represents the concentration of the activated Rab5 with bound GTP, and *x_2_* represents the concentration of the Rabex-5/Rabaptin-5/Rab5-GTP tripartite complex. *x_tot_=x_0_+x_1_+x_2_* is the total amount of Rabex-5, where *x_0_* is the amount of free, cytosolic Rabex-5 and *x_1_*=*σx_0_* is the membrane bound Rabex-5. *σ* is an association constant. Rabex-5 is functional, as a GEF, either on the membrane or in the tripartite complex.

To find the steady state of the system, we set
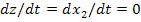
. Then we have from Eqs. (S1) and (S2):


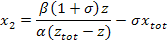

 (S3)


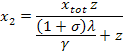

 (S4)

The curve in Eq. (S4) is monotonically increasing: It starts at the origin and reaches the plateau *x_tot_* when *z* tends toward infinity. Because of *z* ≤ *z_tot_*, the condition for *x* to be near *x_tot_* is *z_tot_* >> *(1+σ)λ/γ*. This curve has a negative curvature (Fig S3, A).

On the other hand, the curve in Eq. (S3) is also monotonically increasing. It does not start at the origin (except when *σ* = 0) but at


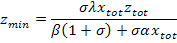

 (S5)

Eventually, it tends toward infinity at the asymptote *z = z_tot_*. This curve has a positive curvature in the range [*z_min_*,*z_tot_*] (Fig. S3, A).

Therefore, there is only one positive intersection between the two curves from Eq. (S3) and Eq. (S4), which is the unique steady state of the system (Fig. S3, A).

For *σ = 0*, there are two possible arrangements between the two curves, which are both monotonically increasing and passing through the origin, but exhibit opposite curvatures. Figs S3B and S3C show the two arrangements. The critical condition for the two cases is that the slopes for the curves from Eq. (S3) and Eq. (S4) are equal at the origin:


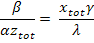


that is


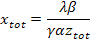

 (S6)

This is exactly the onset condition given in Eq. 7 of the main text.

The system does not have bistability. The feedback is not sufficiently robust in comparison to the model proposed by Xiong and Ferrell (2001). One of the two steady states is always unstable (Bishop and Qian, 2010). The branching is a transcritical bifurcation rather than a saddle-node bifurcation that usually produces bistability (Strogatz, 2001).

Combining Eqs. (S3) and (S4), we have:


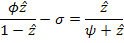

 (S7)

where


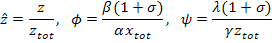

 (S8)

Therefore,


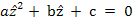

 (S9)

that is


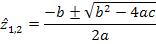

 (S10)

with

*a* = *1 + ϕ + σ*, *b* = *ϕψ + σψ – 1 - σ*, c = -*σψ*. (S11)

Fig. S4 shows
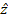
 as a function of *x_tot_*, with *σ* = 0.01, 0.5 and 5. The values for the other parameters, taken from the main text, are:


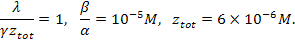

 (S12)

This corresponds to a critical *x_tot_* = 10μM, according to Eq. (S6), where the delayed onset occurs.

References

Ferrell, J. and Xiong, W. (2001) Bistability in cell signaling: How to make continuous processes discontinuous, and reversible processes irreversible. *Chaos,* **11**, 227-236.

Bishop, L.M. and Qian, H. (2010) Stochastic bistability and bifurcation in a mesoscopic signaling system with autocatalytic kinase. *Biophys. J.* **98**, 1-11.

Strogatz, S. (2001) *Nonlinear Dynamics and Chaos: With Applications to Physics, Biology, Chemistry, and Engineering* (Studies in Nonlinearity). Perseus Books Group.

1. Supplemental material for “Delayed Onset of Positive Feedback Activation of Rab5 by Babex-5 and Rabaptin-5 in Endocytosis”, by the same authors, published by *PLoS ONE* (2010). [↑](#footnote-ref-2)
