# Supplementary figures and images for: Delayed Onset of Positive Feedback Activation of Rab5 by Rabex-5 and Rabaptin-5 in Endocytosis
Source: PLoS One. 2010 Feb 16;5(2):e9226. doi: 10.1371/journal.pone.0009226 (PMC2821916; doi:10.1371/journal.pone.0009226)

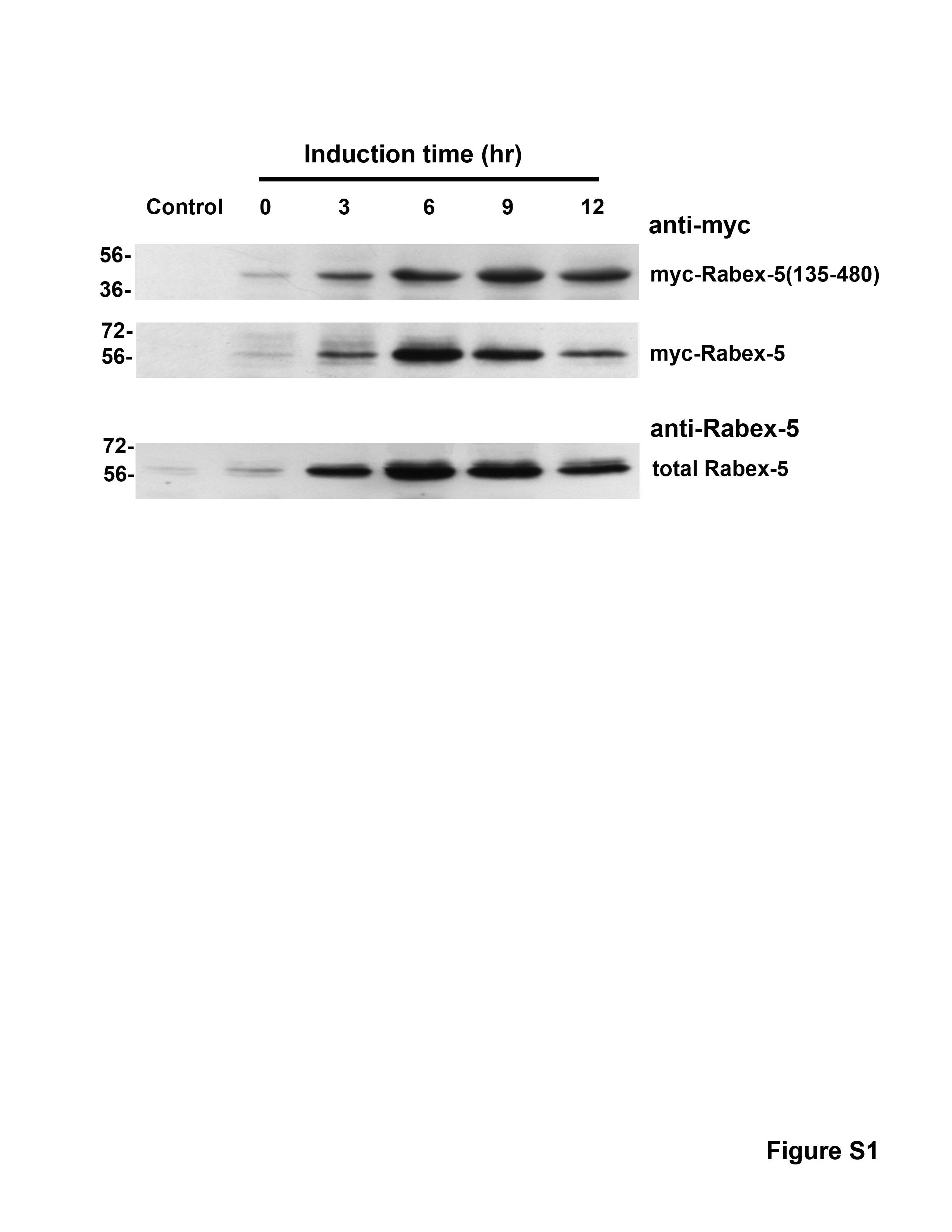

Supplement: Figure S1 — Inducible expression of Rabex-5 constructs in BHK cells. Shown are immunoblots done in parallel with those in Figure 3, indicating the inducible expression of different myc-tagged Rabex-5 constructs without Rabaptin-5. The experiments were the same as that in Figure 3, except pBI/myc-Rabex-5(135–480)/Rabaptin-5 was substituted by pBI/myc-Rabex-5(135–480) and pBI/myc-Rabex-5, respectively, in the transfection as indicated. The expression of each construct was identified by the anti-myc mAb. The full-length myc-Rabex-5 expression was also probed by the anti-Rabex-5 antibody to gauge the level of ectopic expression over endogenous Rabex-5. Molecular mass standards (in kDa) are indicated on the left side of the panel. (0.43 MB TIF) [file pone.0009226.s002.tif]

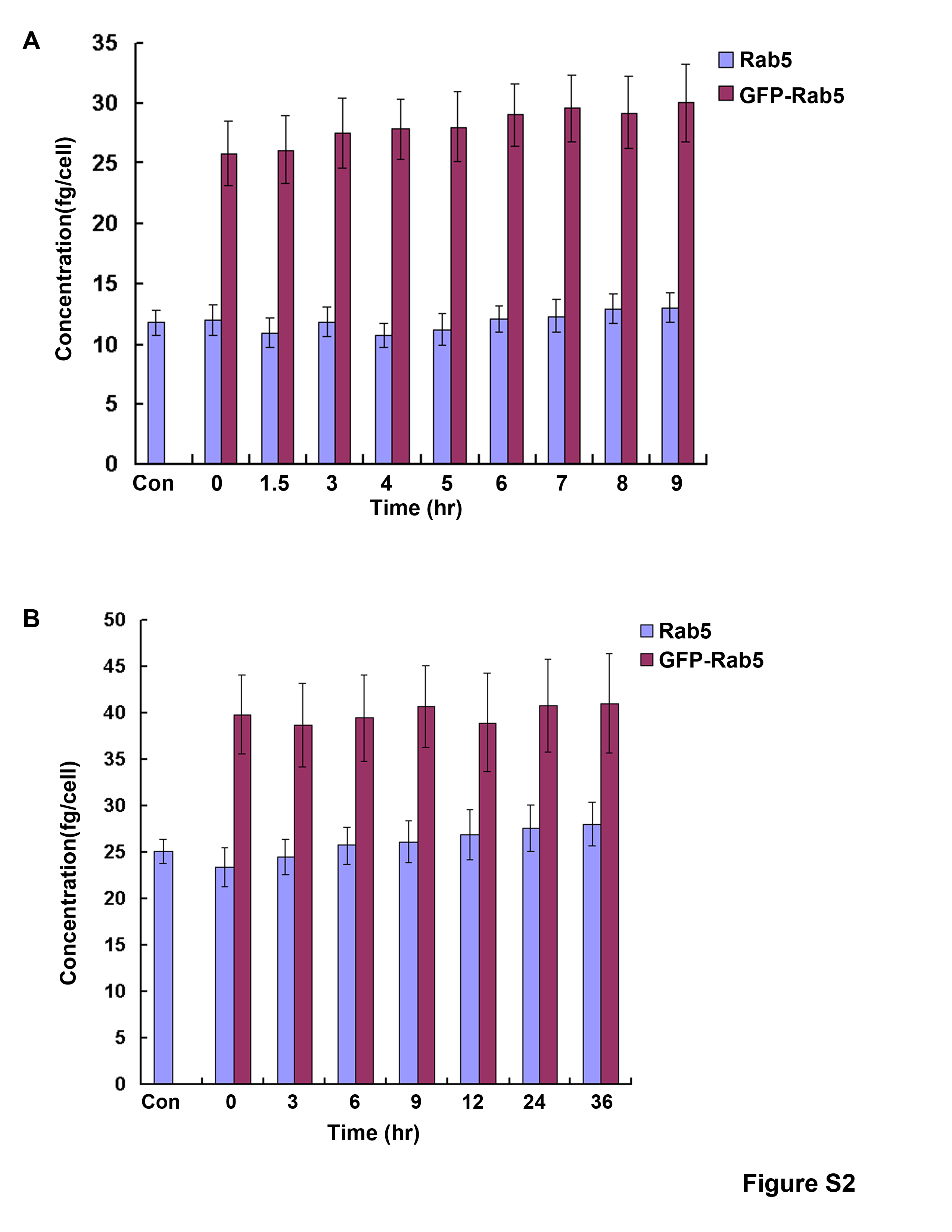

Supplement: Figure S2 — Quantification of endogenous Rab5 and GFP-Rab5 in BHK and NF73 cells. A. Shown are endogenous Rab5 and GFP-Rab5 concentrations in BHK cells. The quantification was conducted by immunoblot analysis with an anti-Rab5 mAb (see Figure 3), by comparison with a standard curve generated with known concentrations of recombinant Rab5, as described in Materials and Methods. The values for GFP-Rab5 were corrected by transfection efficiency of BHK cells (80%). In the text, the values of 12 fg/cell (Rab5) and 25 fg/cell (GFP-Rab5) were used to convert to molar concentrations and both were determined to be 6×10^−6^ M considering their difference in molecular weight. B. Shown are endogenous Rab5 and GFP-Rab5 concentrations in NF73 cells. The quantification was conducted as described above (see Figure 7). The values for GFP-Rab5 were corrected by transfection efficiency of NF73 cells (20%). In the text, the values of 25 fg/cell (Rab5) and 40 fg/cell (GFP-Rab5) were used to convert to molar concentrations and both were determined to be 1.2×10^−5^ M considering their difference in molecular weight. (1.68 MB TIF) [file pone.0009226.s003.tif]

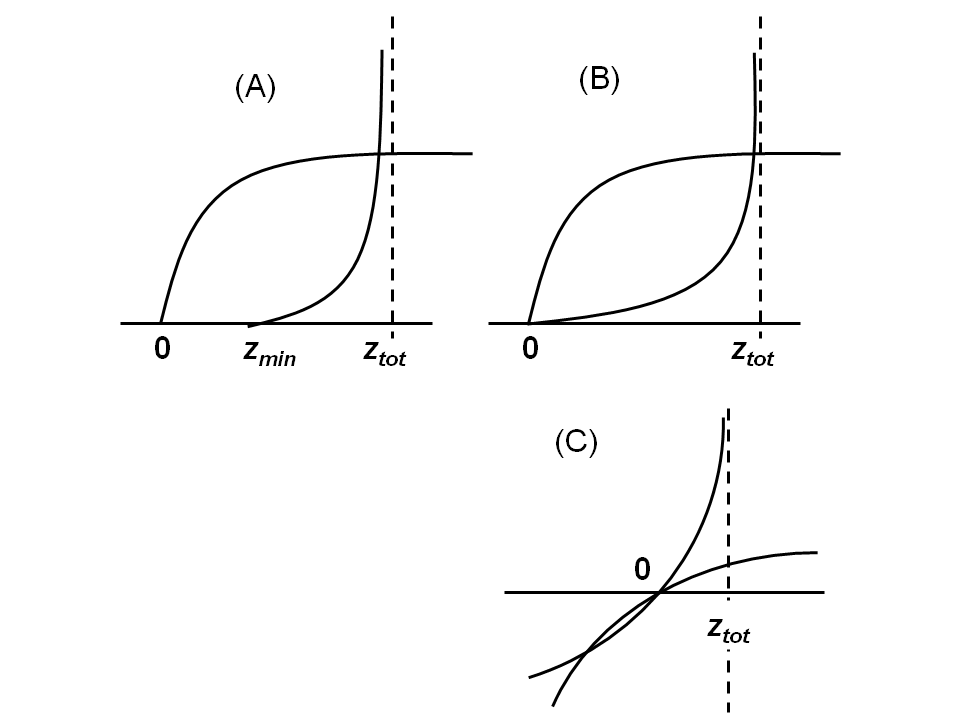

Supplement: Figure S3 — A. Two curves in Eqs. (S3) and (S4), with σ > 0. B. The two curves in Eqs. (S3) and (S4), with σ = 0, have a unstable steady state at the origin and a stable positive steady state. This requires xtot > λβ/(γαztot). C. The two curves in Eqs. (S3) and (S4), with σ = 0, has a negative steady state that requires xtot < λβ/(γαztot). In this case, the zero steady state is stable. (2.77 MB TIF) [file pone.0009226.s004.tif]

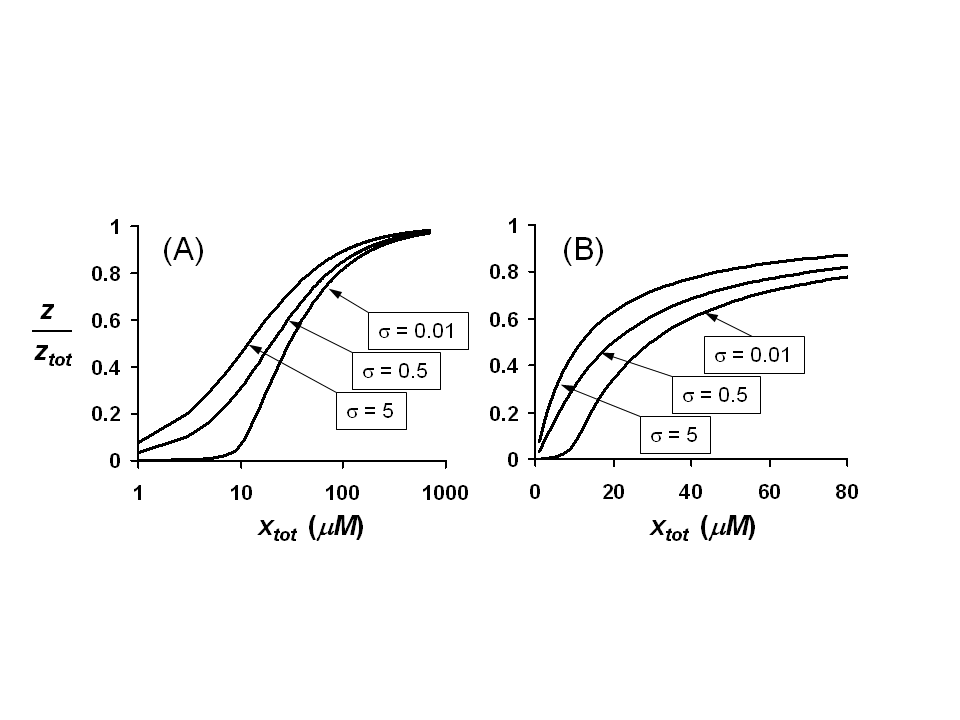

Supplement: Figure S4 — z/ztot as a function of xtot according to Eq. (S10) with the parameters given in Eq. (S12). A. Abscissa in terms of logarithmic xtot. B. Abscissa in terms of linear xtot. The activation curve is hyperbolic for large σ and sigmoidal for small σ. The delayed onset occurs at xtot = 10. (0.07 MB TIF) [file pone.0009226.s005.tif]
